# Supplementary figures and images for: RD5-mediated lack of PE_PGRS and PPE-MPTR export in BCG vaccine strains results in strong reduction of antigenic repertoire but little impact on protection
Source: PLoS Pathog. 2018 Jun 18;14(6):e1007139. doi: 10.1371/journal.ppat.1007139 (PMC6023246; doi:10.1371/journal.ppat.1007139)

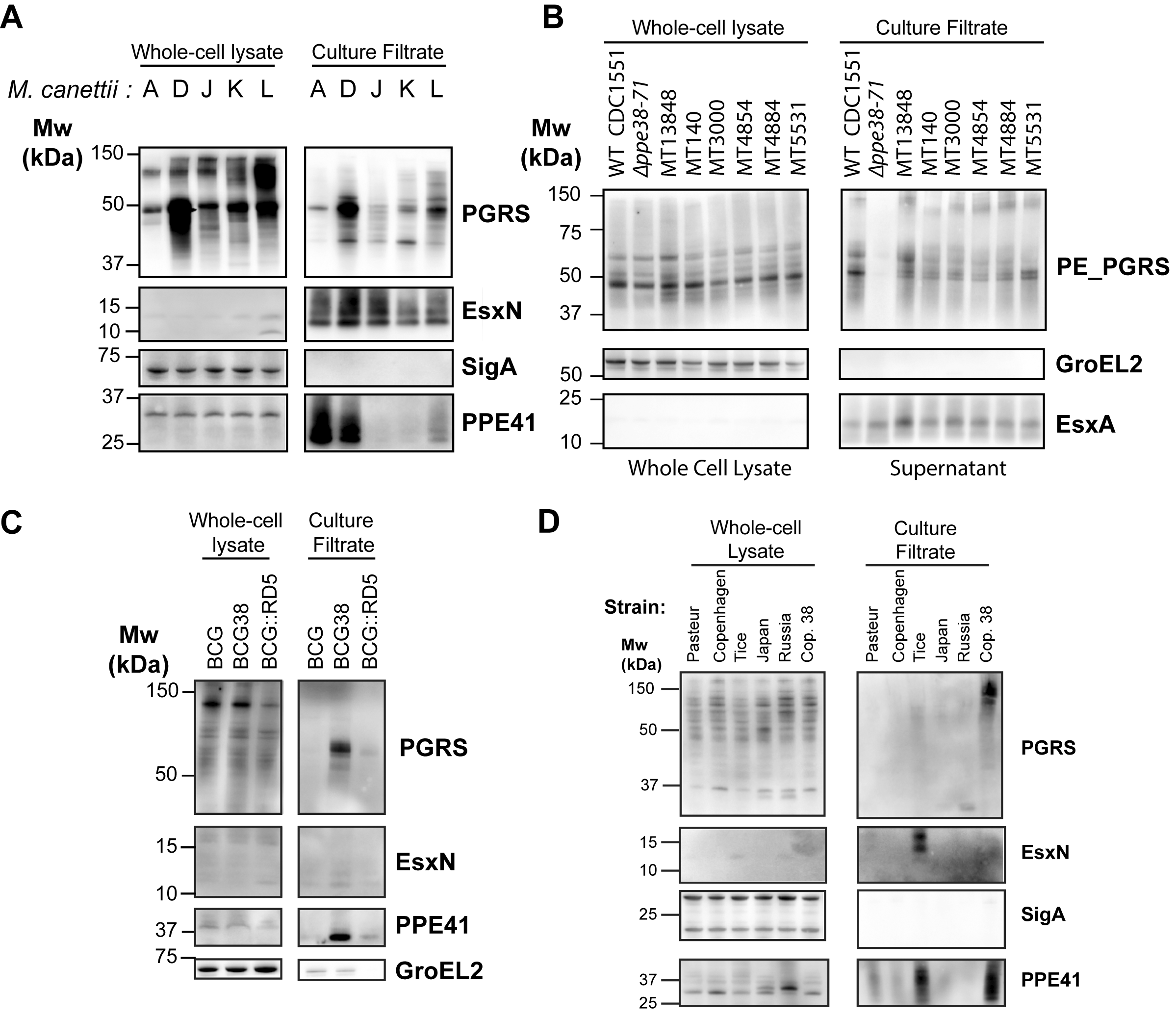

Supplement: S1 Fig — Immunoblots of whole-cell lysates or culture filtrates of the indicated M. canetti (A), M. tuberculosis (B) or BCG (C, D) isolates [48,51]. A) Although differences in protein secretion could be observed between different M. canetti isolates (A-J), all isolates exhibited PE_PGRS secretion. B) PE_PGRS secretion of Mj-sublineage strains with a deletion affecting ppe38, but not ppe71 (Lanes 4–8) was not discernible from Lineage 4 control isolate CDC1551 or an isolate from the same cohort without this deletion (MT13848). C) Introduction of plasmid pMV::ppe38-71 in BCG complemented PE_PGRS secretion (BCG38), while complementation was not observed when performed with pYUB::RD5, even though presence of genetic presence of RD5 was PCR-confirmed with primers RD5B-plcA.int.F/R [52]. D) Immunoblot secretion analysis of five genetically divergent BCG isolates confirms the PE_PGRS secretion defect in all BCG isolates. Cop. 38 indicates the strain M. bovis BCG Copenhagen, transformed with vector pMV::ppe38-71 and is hereafter referred to as BCG38. Anti-SigA staining is uses as a lysis control in A and D, while anti-GroEL2 is used in B and C. Strain details can be found in S5 Table. Full blots of panels A-D are depicted in S5 Fig and S6 Fig. (TIF) [file ppat.1007139.s001.tif]

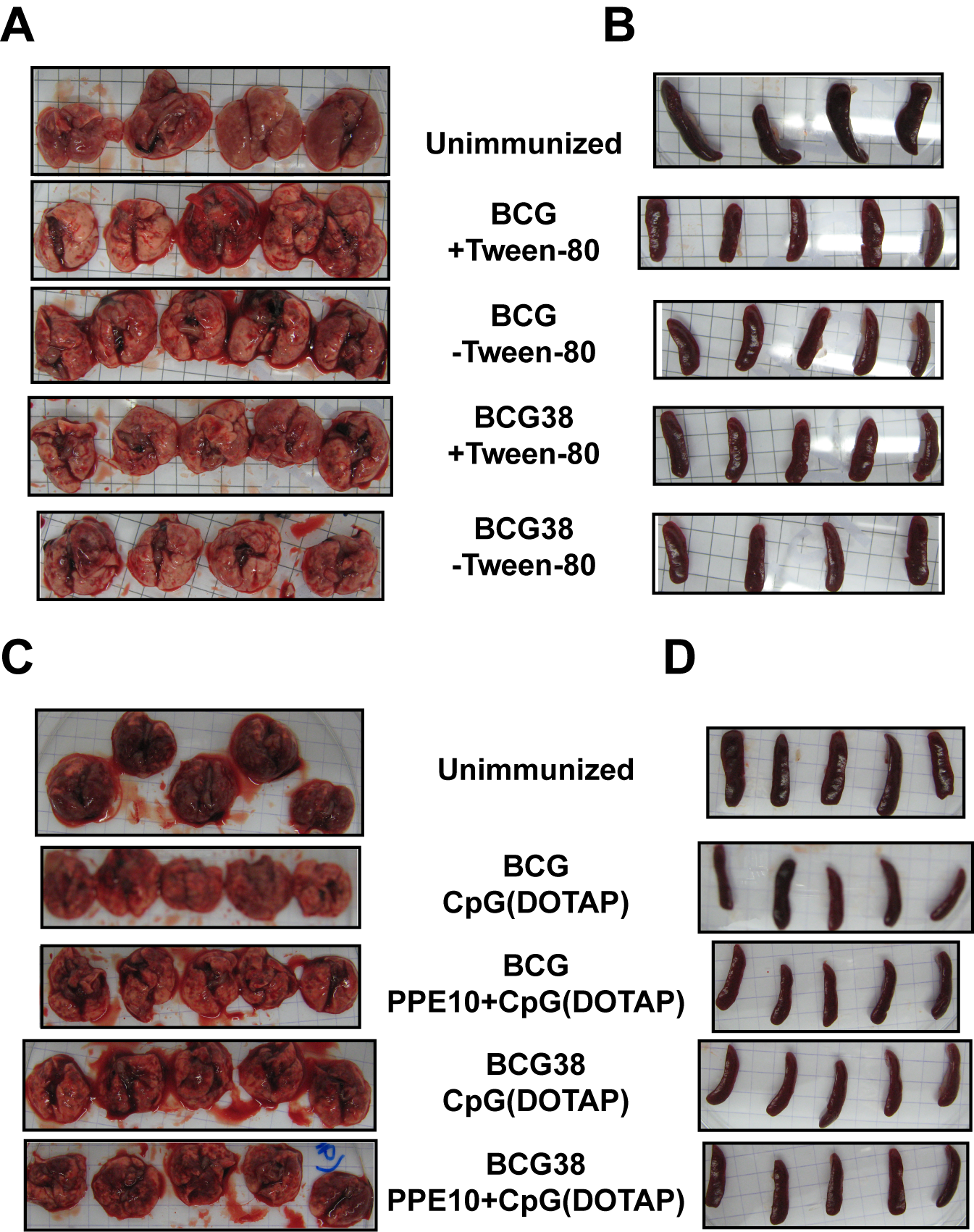

Supplement: S2 Fig — Organs depicted in A and B correspond to the experiment depicted in Fig 3. Organs depicted in C and D correspond to the experiment depicted in Fig 6. After photography of the lungs (A, C), a single lung lobe was used for lung CFU quantification. Splenomegaly (B, D) was reduced, by all vaccination conditions, but did not differ markedly between vaccination conditions. (TIF) [file ppat.1007139.s002.tif]

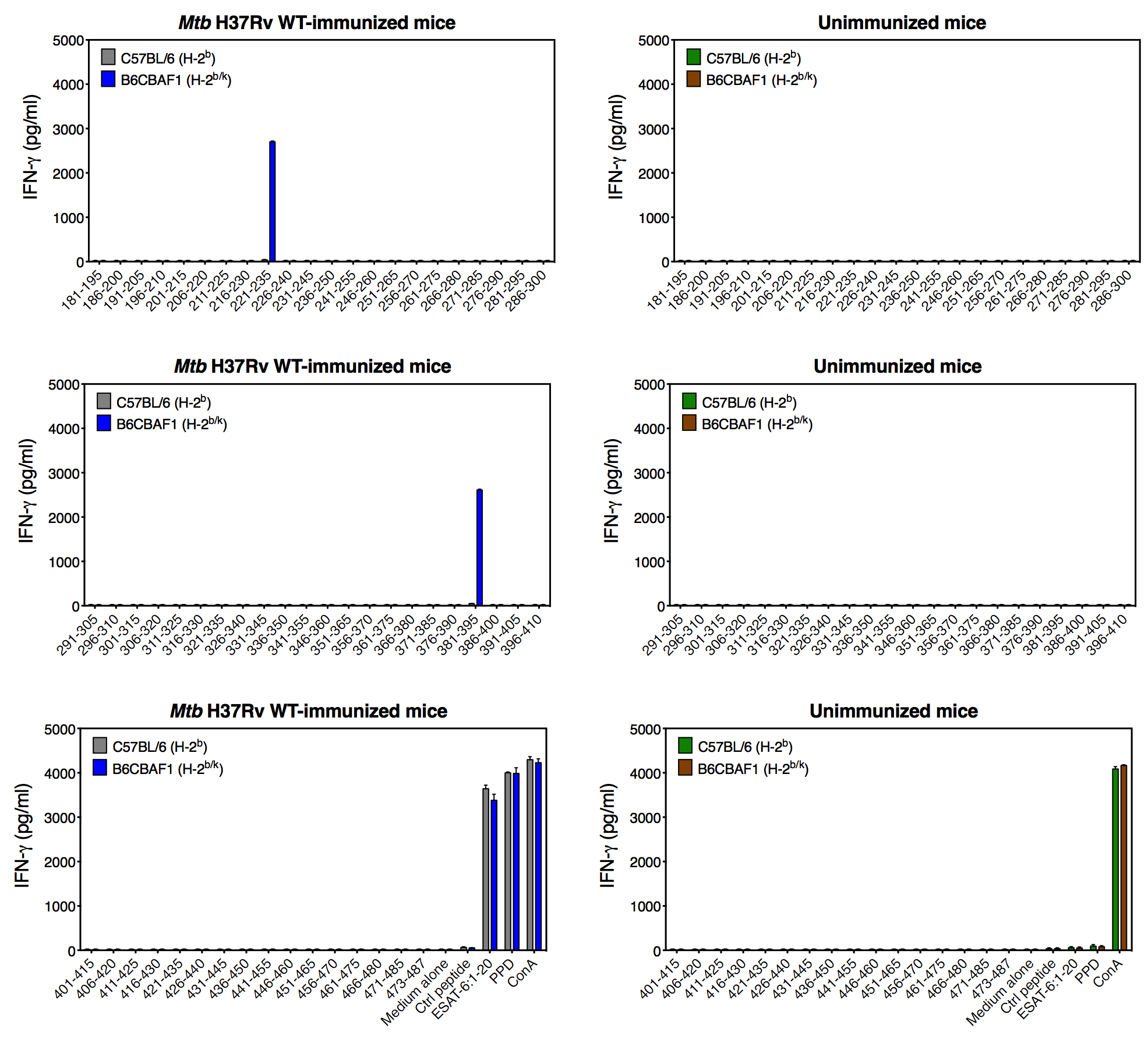

Supplement: S3 Fig — IFN-γ production in response to peptides covering the indicated amino acid positions of PPE10 (Rv0442c) in C57BL/6 (grey/green) or C57BL/6 x CBA F1 (B6CBAF1, blue/brown) mice. Mice were immunized with M. tuberculosis H37Rv (left) or unimmunized (right). (TIF) [file ppat.1007139.s003.tif]

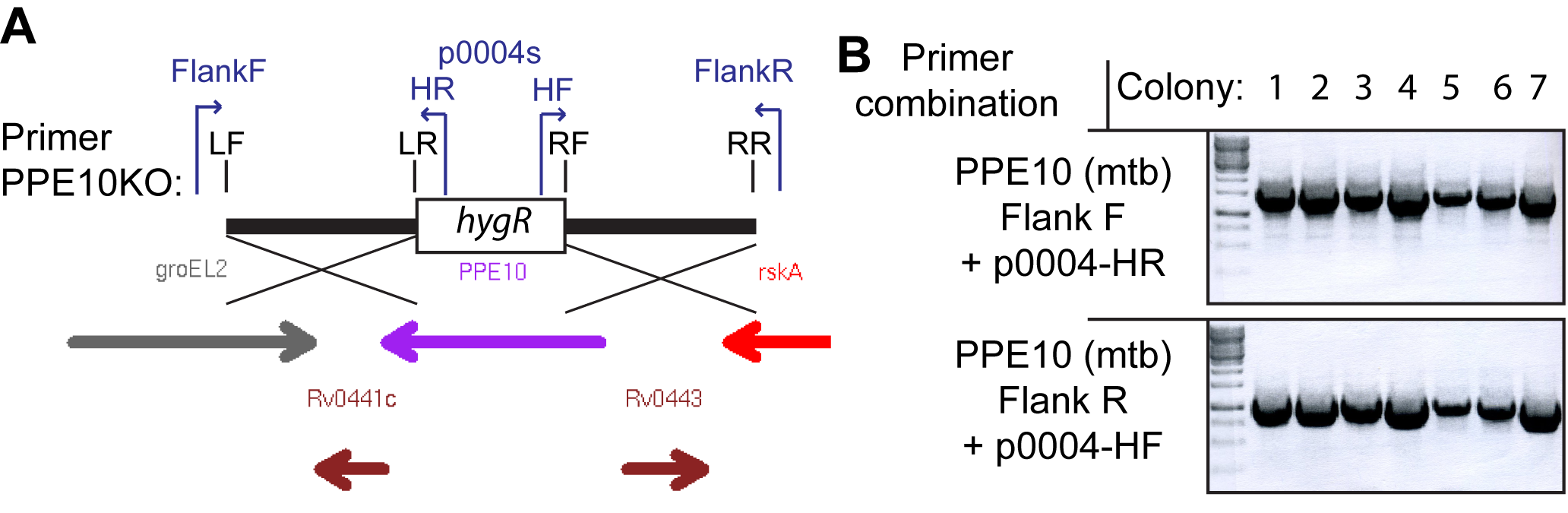

Supplement: S4 Fig — A) Schematic representation of deletion strategy and primers. The genetic region around PPE10, as taken from tuberculist, is depicted in colored arrows [74]. Flanking fragments used for homologous recombination are depicted in black bars. Left (PPE10KO-LF and PPE10KO-LR) and right (PPE10KO-RF and PPE10KO-RR) flanking regions were amplified by primers depicted in black. Primers used to verify successful homologous recombination are depicted in dark blue. All primer sequences can be found in S4 Table. B) PCR verification of successful homologous recombination in seven different colonies that grew on hygromycin selection plates. Colony 1 was taken for further analyses. Full gels used to create B are depicted in S7 Fig. (TIF) [file ppat.1007139.s004.tif]

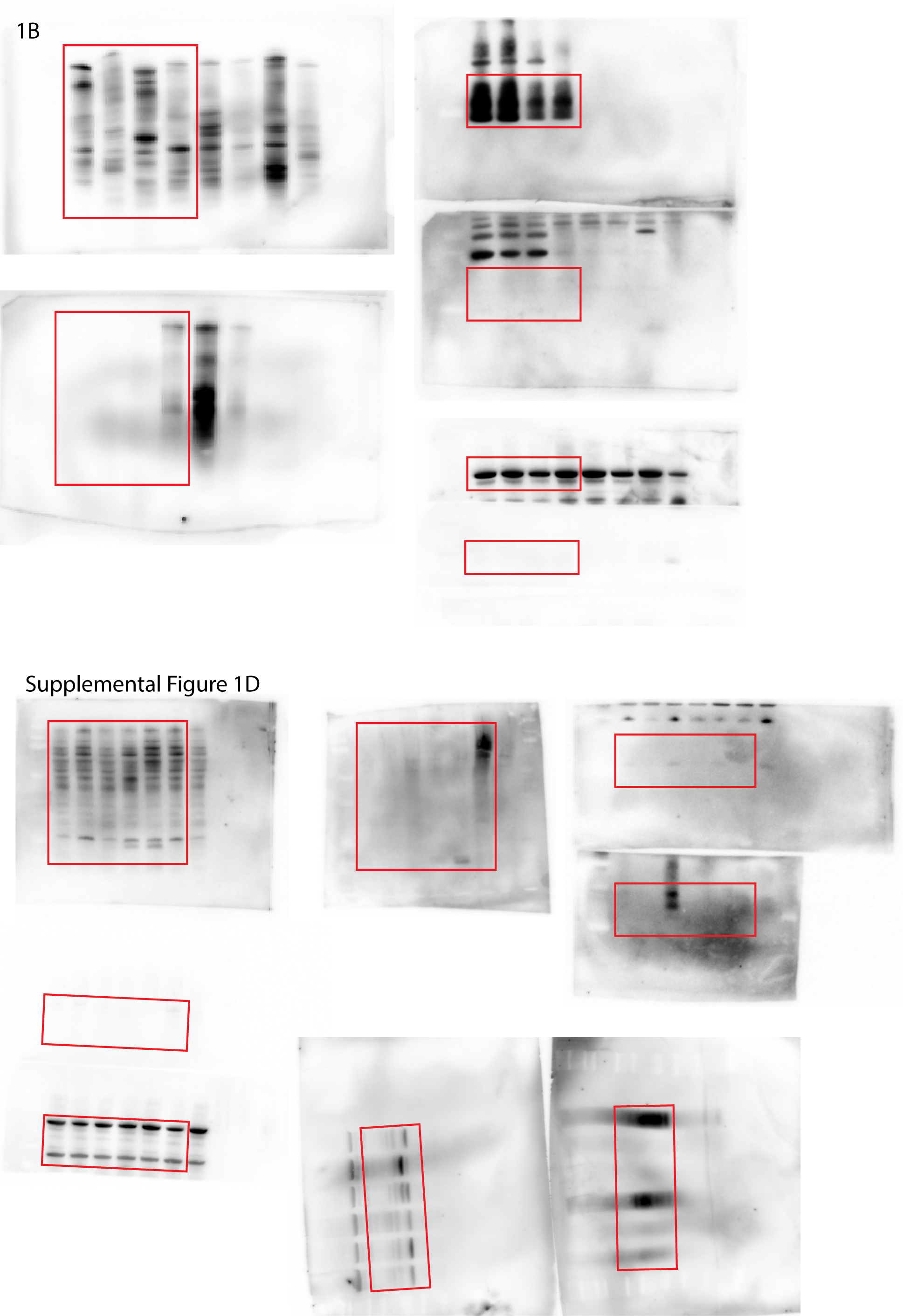

Supplement: S5 Fig — (TIF) [file ppat.1007139.s005.tif]

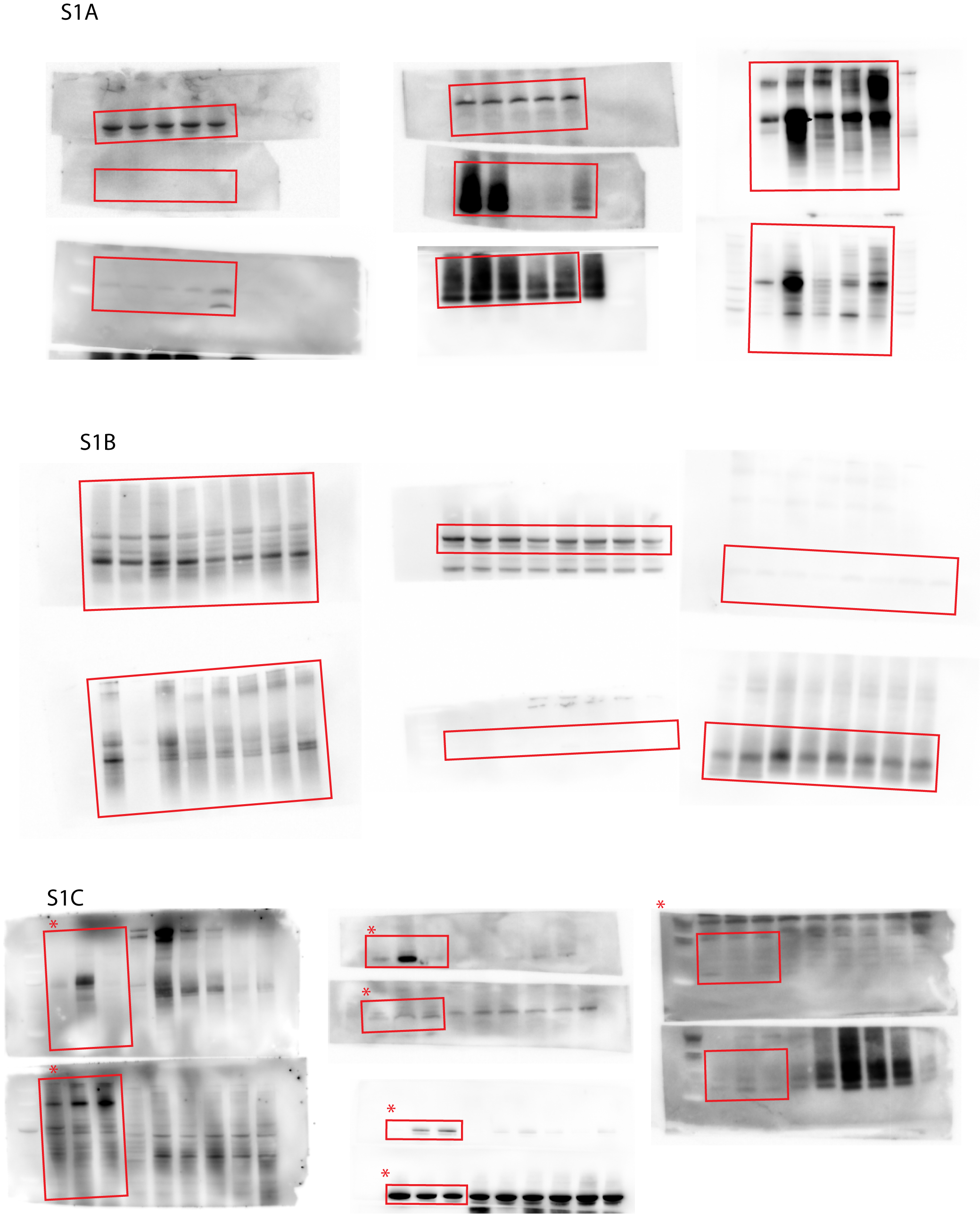

Supplement: S6 Fig — (TIF) [file ppat.1007139.s006.tif]

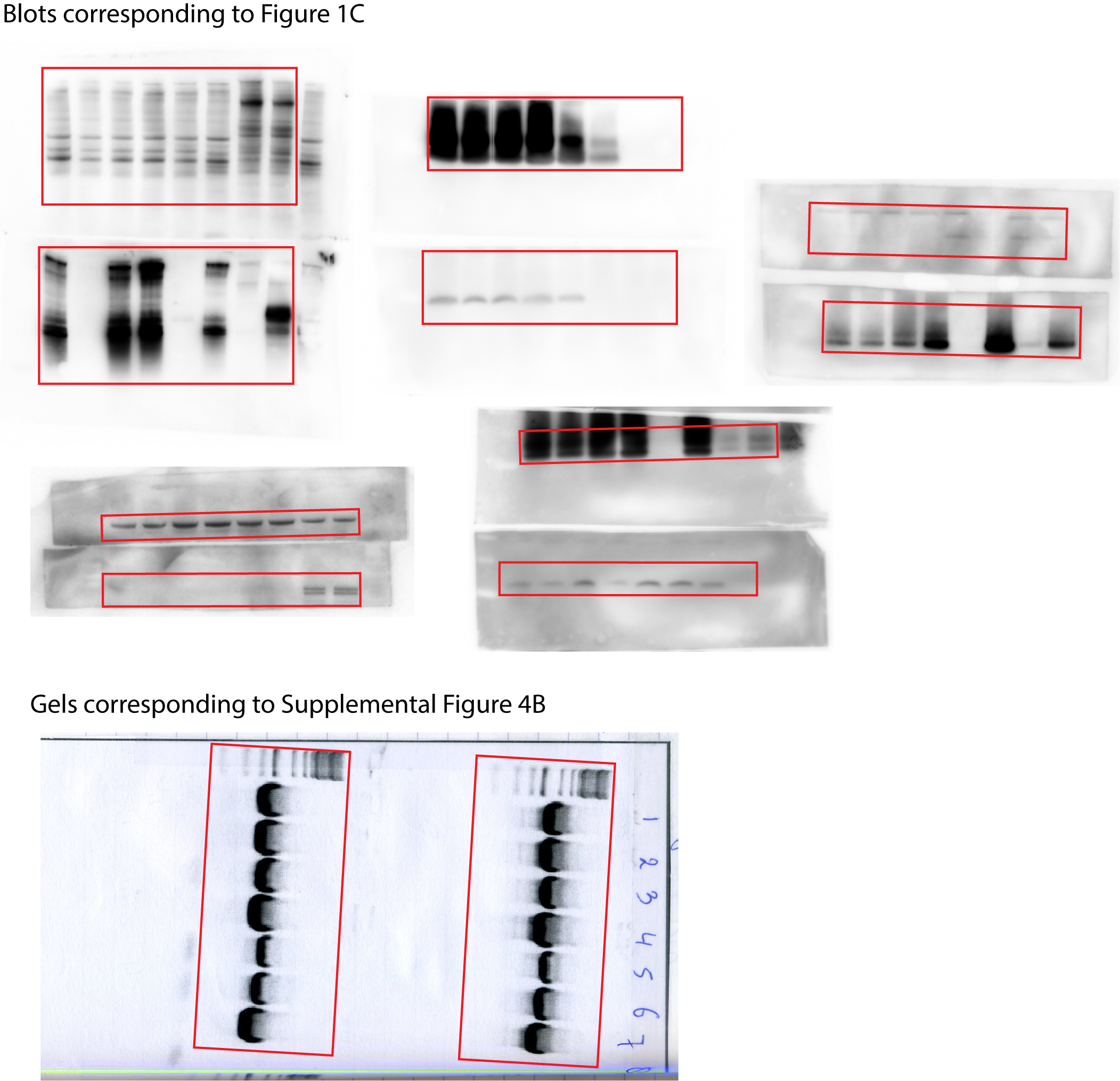

Supplement: S7 Fig — (TIF) [file ppat.1007139.s007.tif]
