# Supplementary material for: RD5-mediated lack of PE_PGRS and PPE-MPTR export in BCG vaccine strains results in strong reduction of antigenic repertoire but little impact on protection
Source: PLoS Pathog. 2018 Jun 18;14(6):e1007139. doi: 10.1371/journal.ppat.1007139 (PMC6023246; doi:10.1371/journal.ppat.1007139)
Supplement: S1 Table — As expected, the percentage of live cells was higher for BCG-infected cells than for cells infected with M. tuberculosis strains, but did not vary significantly between isogenic strains (≤ 1.0% difference between isogenic strain). These values are derived from the experiment depicted in Fig 2A. (PDF) [file ppat.1007139.s008.pdf]

	CD40	CD80	CD86	H-2K^b	I-A^b	% Live Cells
**Uninfected**	218	338	264	663	904	94.9
**CDC1551**	282	449	427	982	1404	82.5
**Δ*ppe38-71***	287	424	446	925	1360	81.7
***ppe38-71*-C**	303	457	454	969	1419	81.5
**BCG**	273	430	390	810	1348	86.6
**BCG38**	276	429	381	832	1417	86.8

S1 Table. Mean Fluorescent Intensities and percentage of live C57BL/6 BM-DCs, infected (MOI = 0.5) with indicated strains of *M. tuberculosis* or *M. bovis* BCG with or without the *ppe38*-locus. As expected, the percentage of live cells was higher for BCG-infected cells than for cells infected with *M. tuberculosis* strains, but did not vary significantly between isogenic strains (≤ 1.0 % difference between isogenic strain). These values are derived from the experiment depicted in Fig 2A.
