# Supplementary material for: RD5-mediated lack of PE_PGRS and PPE-MPTR export in BCG vaccine strains results in strong reduction of antigenic repertoire but little impact on protection
Source: PLoS Pathog. 2018 Jun 18;14(6):e1007139. doi: 10.1371/journal.ppat.1007139 (PMC6023246; doi:10.1371/journal.ppat.1007139)
Supplement: S2 Table — A promoter mutation disrupts production of I-Eαb in C57BL/6 mice (Grey font), which are therefore unable to produce MHC-II I-E (Grey). In contrast, H-2k mice can produce both I-Aαk I-Aαk and I-Eαk I-Eαk. C57BL/6 x CBA F1 mice have an even bigger repertoire of possible functional MHC-II isoforms available due to heterodimerization of α and β subunits from H-2b and H-2k haplotypes. (PDF) [file ppat.1007139.s009.pdf]

	MHC-II	MHC-II	MHC-II	MHC-II
**MHC Haplotype H-2^{b}** (C57BL/6 mice)	I-A_{α}^{b}	I-A_{β}^{b}	(I-E_{α}^{b} not present)	I-E_{β}^{b}
**Functional MHC-II available in H-2^{b}**	I-A_{α}^{b} I-A_{β}^{b}		No I-E available in H-2^{b}	
**MHC Haplotype H-2^{k}** (CBA mice)	I-A_{α}^{k}	I-A_{β}^{k}	I-E_{α}^{k}	I-E_{β}^{k}
**Functional MHC-II available in H-2^{k}**	I-A_{α}^{k} I-A_{β}^{k}		I-E_{α}^{k} I-E_{β}^{k}	
**H-2$^{b/k}$** (C57BL/6 x CBA)	I-A_{α}^{b}<br>I-A_{α}^{k}	I-A_{β}^{b}<br>I-A_{β}^{k}	(I-E_{α}^{b} not present)<br>I-E_{α}^{k}	I-E_{β}^{b}<br>I-E_{β}^{k}
**Functional MHC-II available in H-2 $^{b/k}$**	Not only	I-A_{α}^{b} I-A_{β}^{b}	I-A_{α}^{k} I-A_{β}^{k}	I-E_{α}^{k} I-E_{β}^{k}
	But also	I-A_{α}^{b} I-A_{β}^{k}	I-A_{α}^{k} I-A_{β}^{b}	I-E_{α}^{k} I-E_{β}^{b}

S2 Table. Why there are more variants of MHC-II restricting molecules available in C57BL/6 x CBA F1 mice than in C57BL/6 or CBA mice? A promoter mutation disrupts production of I-Eα^{b} in C57BL/6 mice (Grey font), which are therefore unable to produce MHC-II I-E (Grey). In contrast, H-2^{k} mice can produce both I-Aα^{k} I-Aα^{k} and I-Eα^{k} I-Eα^{k}. C57BL/6 x CBA F1 mice have an even bigger repertoire of possible functional MHC-II isoforms available due to heterodimerization of α and β subunits from H-2^{b} and H-2^{k} haplotypes.
