# Supplementary material for: RD5-mediated lack of PE_PGRS and PPE-MPTR export in BCG vaccine strains results in strong reduction of antigenic repertoire but little impact on protection
Source: PLoS Pathog. 2018 Jun 18;14(6):e1007139. doi: 10.1371/journal.ppat.1007139 (PMC6023246; doi:10.1371/journal.ppat.1007139)
Supplement: S3 Table — Black letters indicate identical amino acids. Red letters indicate non-identical amino acids. Top: homologues of the MPTR-containing peptide PPE10221-235 ordered by percentage of sequence identity. Bottom: Homologues of the peptide PPE10381-395, which is part of the C-terminal secreted domain of PPE10. (PDF) [file ppat.1007139.s010.pdf]

**Epitope PPE10$_{221-235}$**	**Protein Number**	**Amino acid Sequence Identity**	**Sequence identity (100%)**
PPE10	Rv0442c	GSGNTGSGNLGLGNL	100
PPE13	Rv0878c	GSGNEGSGNLGFGNL	86.7
PPE39	Rv2353c	GFGNTGSGNFGFGNT	80.0
PPE53	Rv3159c	GSGNTGSTNFGGGNL	80.0
PPE16	Rv1135c	GSGNDGNGNFGLGNI	73.3
PPE21	Rv1548c	GSGNLGSGNIGFGNK	73.3
PPPE56	Rv3350c	GLGNVGDGNLGLGNI	73.3
PPE40	Rv2356c	GFGNTGSGNFGFGNT	73.3
PPE55	Rv3347c	GSGNVGFGNMGVGNI	66.7
PPE34	Rv1917c	GIGNTGTGNFGIGNS	66.7
PPE6	Rv0305c	GIGNSGTGNFGLGNT	66.7
PPE8	Rv0355c	GIGNTGTGNIGFGNT	66.7
PPE5	Rv0304c	GIGNTGTGNFGIGNS	66.7
PPE62	Rv3533c	GTGNAGSGNIGAGNT	66.7
PPE54	Rv3343c	GSGNVGSYNVGAGNV	66.7
PPE24	Rv1753c	GFGNLGSNNVGVGNL	66.7
PPE42	Rv2608	ASGNLGSGNVGVGNI	66.7
PPE64	Rv3558	GAGNVGTGNIGFGNQ	60.0
PPE35	Rv1918	GIGNAGANNFGLANL	60.0

**Epitope PPE10$_{381-395}$**	**Protein Number**	**Amino acid Sequence Identity**	**Sequence identity (100%)**
PPE10	Rv0442c	NVLNSGLTNTPVAAP	100
PPE12	Rv0755c	GFLNSGLTNTGFANS	60.0
PPE8	Rv0355c	GLLNAGLVNTGIANP	53.3

S3 Table. Sequence identity determined by BlastP search of immunogenic epitopes against the genome of *M. tuberculosis* H37Rv [29,106]. Black letters indicate identical amino acids. Red letters indicate non-identical amino acids. Top: homologues of the MPTR-containing peptide PPE10$_{221-235}$ ordered by percentage of sequence identity. Bottom: Homologues of the peptide PPE10$_{381-395}$, which is part of the C-terminal secreted domain of PPE10.
