# Supplementary material for: RD5-mediated lack of PE_PGRS and PPE-MPTR export in BCG vaccine strains results in strong reduction of antigenic repertoire but little impact on protection
Source: PLoS Pathog. 2018 Jun 18;14(6):e1007139. doi: 10.1371/journal.ppat.1007139 (PMC6023246; doi:10.1371/journal.ppat.1007139)
Supplement: S4 Table — (PDF) [file ppat.1007139.s011.pdf]

Primer name	Sequence 5'→3'
RD5B-plcA.int.F	CAAGTTGGGTCTGGTCGAAT [52]
RD5B-plcA.int.R	GCTACCCAAGGTCTCCTGGT [52]
PPE38F	TTTTCGGTGTGGATTGTCT [38]
PPE38R	CCAGGGATTTCCAACGAC [38]
PPE10 KO LF	TTTTTTTTCAG**CTT**CTGACCGGCGCCAACATCGTGAA
PPE10 KO LR	TTTTTTTTCAG**AGA**CTGCCTGGCGAACGTCCTCAACT
PPE10 KO RF	TTTTTTTTCAG**TTC**CTGACGGAGCCAAGCGACGCTAT
PPE10 KO RR	TTTTTTTTCAG**AAA**CTGCTCGACCGCACTGGCATTCA
PPE10(mtb) flank F	GAACAGCGACTCCGACTACG
PPE10(mtb) flank R	CTCGACCGCACTGGCATTCA
p0004s-HL	AGGATCCAGGACCTGCCAAT
p0004s-HR	CTTCACCGATCCGGAGGAAC

S4 Table. Primers used in this study
