# Supplementary material for: RD5-mediated lack of PE_PGRS and PPE-MPTR export in BCG vaccine strains results in strong reduction of antigenic repertoire but little impact on protection
Source: PLoS Pathog. 2018 Jun 18;14(6):e1007139. doi: 10.1371/journal.ppat.1007139 (PMC6023246; doi:10.1371/journal.ppat.1007139)
Supplement: S5 Table — (PDF) [file ppat.1007139.s012.pdf]

Species	Strain code	reference	Notes
Mycobacterium canettii	STB-A	[48]	
Mycobacterium canettii	STB-D	[48]	
Mycobacterium canettii	STB-J	[48]	
Mycobacterium canettii	STB-K	[48]	
Mycobacterium canettii	STB-L	[48]	
Mycobacterium bovis	CVL AF2122/97	http:// genolist.pasteur.fr/ BoviList	
Mycobacterium caprae	140080001		Pasteur strain collection
Mycobacterium orygis	802564	[56]	Pasteur strain collection
Mycobacterium pinnipedii	140090001		Pasteur strain collection
Mycobacterium bovis BCG	BCG Pasteur	[5,6]	
Mycobacterium bovis BCG	BCG Danish	[5,6]	
Mycobacterium bovis BCG	BCG Tice	[5,6]	
Mycobacterium bovis BCG	BCG Tokyo	[5,6]	
Mycobacterium bovis BCG	BCG Russia	[5,6]	
Mycobacterium bovis BCG	BCG38	This study	BCG Danish - pMV::*ppe38-71*
CDC1551			
CDC1551 Δ*mt2419-22*	Δ*ppe38-71*	[37]	
CDC1551 Δ*mt0458*	Δ*ppe10*	This study	
CDC1551 Δ*mt2419-22,* pMV::*ppe38-71*	Δ*ppe38-71*-C	[37]	Δ*ppe38-71* -pMV::*ppe38-71*
CDC1551 *eccC₅::tn*	*eccC₅::tn*	BEI resources & [57]	BEI resources strain: JHU1783-2086 / Transposon Mutant 1291 (MT1844, Rv1795) (BEI ID NR-14751)
H37Rv wild-type			Reference strain
H37Rv *Δppe25-pe19*	*Δppe25-pe19*	[76]	
MT13848		[51]	Clinical strain, Sublineage Mn
MT5531		[51]	Clinical strain, Sublineage Mj-III.a
MT4854		[51]	Clinical strain, Sublineage Mj-III.b
MT140		[51]	Clinical strain, Sublineage Mj-IV.c
MT4884		[51]	Clinical strain, Sublineage Mj-V.a
MT3000		[51]	Clinical strain, Sublineage Mj-V.c
BCG::RD5		[16]	BCG Pasteur containing cosmid pYUB::RD5

S5 Table. Bacterial strains used in this study
